# Supplementary material for: Control of structural flexibility of layered-pillared metal-organic frameworks anchored at surfaces
Source: Nat Commun. 2019 Jan 21;10:346. doi: 10.1038/s41467-018-08285-5 (PMC6341086; doi:10.1038/s41467-018-08285-5)
Supplement: Supplementary file 1 — Supplementary Information [file 41467_2018_8285_MOESM1_ESM.pdf]

Supplementary Information

Control of Structural Flexibility of Layered-Pillared Metal-Organic Frameworks Anchored at Surfaces

Wannapaiboon, S. *et. al.*

1. Supplementary Methods

Materials. All reagents and solvents were purchased from commercial suppliers (Sigma Aldrich, TCI and Alfar Aesar) and used without further purification. $Cu(NO_3)_2{\cdot}3H_2O$ and $Cu(OAc)_2{\cdot}H_2O$ were used as metal precursors for powder synthesis and thin-film fabrication of the Cu-based layered-pillared metal—organic frameworks (MOFs), respectively. The alkoxy-functionalised linker, i.e. 2,5-diethoxy-1,4-benzenedicarboxylic acid (H_2DE-bdc) and 2,5-bis(2-methoxyethoxy)-1,4-benzenedicarboxylic acid (H_2BME-bdc), were prepared via Williamson ether synthesis from dimethyl 2,5-dihydroxy-1,4-benzenedicarboxylate according to published procedure.[1] 1,4-diazabicyclo[2.2.2]octane (dabco) was used as a pillared linker (see Supplementary Figure 1).

H_2DE-bdc; ^{1}H-NMR (200 MHz, DMSO-d6): δ 12.90 (s, 2H), 7.26 (s, 2H), 4.04 (d, 4H), 1.29 (t, 6H).

H_2BME-bdc; ^{1}H NMR (250 MHz, DMSO-d6): δ 7.30 (s, 1H), 4.12 (dd, J = 5.3, 3.9 Hz, 2H), 3.64 (dd, J = 5.3, 3.9 Hz, 2H), 3.31 (s, 3H).

Bulk powder MOF synthesis. Cu_2(DE-bdc)$_2$(dabco) powder (**1bulk**) was prepared under solvothermal reaction condition, which was slightly modified from the previously reported procedure for the Zn^{2+} containing analogue.[1] $Cu(NO_3)_2{\cdot}3H_2O$ (241.6 mg, 1 mmol), H_2DE-bdc (254.2 mg, 1mmol) and dabco (56.0 mg, 0.5 mmol) were suspended in DMF (15 ml) and sonicated until the precursors were fully dissolved. The solution was left at room temperature for 20 min and then a precipitate formed. This precipitate was removed by filtration prior to transferring the clear filtrate into a screw jar (25 ml), which was sealed and subsequently heated to 120 °C for 48 h. After cooling down to room temperature, the mother liquor was firstly decanted and then exchanged by fresh DMF, the mixture was stirred for 30 min and left to settle for 24 h. Afterwards, the DMF was exchanged by $CHCl_3$, the mixture was stirred for 30 min and left to settle for 24 h.

The solvent exchange procedure with $CHCl_3$ was repeated for 2 times prior to collecting **1bulk** by filtration with a frit. The collected product was washed 3 more times with 10 ml $CHCl_3$. **1bulk** was subsequently transferred to a Schlenk tube and activated overnight at 130 °C. Cu_2(BME-bdc)$_2$(dabco) powder (**2bulk**) was prepared using the same synthetic procedure as for preparing **1bulk**, instead of H_2DE-bdc, H_2BME-bdc (314.3 mg, 1mmol) was used as a linker.

Characterisations of MOF powders. Crystallinity and phase purity of the compounds **1bulk** and **2bulk** was identified by powder X-ray diffraction (XRD, D8 Bruker-AXS advance instrument, flat mode, Debye-Scherrer geometry, slit width of 0.05°, Cu K_α radiation, 2θ range from 5° to 50°, position sensitive detector, Ni filter and step size of 0.0141°). Infrared (IR) spectra were recorded on a Bruker Alpha-P FT-IR (ATR-Mode, 48 scans) located in a glovebox. Thermogravimetric analysis (TGs) were recorded on a Netzsch STA 409 PC TG-DSC apparatus (heating rate of 5 K min^{-1}, in a stream of N_2 gas with constant flow rate of 20 ml min^{-1}). Roughly 10 mg of samples were placed in a pre-weighted, clean aluminium oxide crucible. The TG curves were background-corrected by subtraction of a measurement conducted with an empty crucible under the same conditions.

MOF thin-film fabrication. Au-coated quartz crystal microbalance sensors (QCM, commercially available by Q-Sense, AT cut type, Au electrode, diameter 14 mm, thickness 0.3 mm and fundamental frequency ca. 4.95 MHz) were used as the substrates. Prior to the fabrication of MOF thin-films, the substrates were immersed in 20 μM solution of 16-mercaptohexadecanoic acid (MHDA) in ethanol mixed with 5% v/v acetic acid for 24 h to generate the COOH terminated substrate. In order to generate the pyridyl terminated surface, 20 μM solution of 4-(4-pyridyl)phenylmethylthiol (PMBT) in ethanol was used instead of MHDA. These substrates were subsequently used for the fabrication of MOF films. Thin-films of **1** and **2** (coined the terms as **1tf$_x$** and **2tf$_x$**; x = total deposition cycles, respectively) were fabricated by a stepwise liquid-phase epitaxial process (LPE, Supplementary Figure 2) at controlled temperature of 40 °C using an automated QCM instrument (Q-Sense E4 Auto) operated in the continuous flow mode with a constant flow rate of 100 μL min^{-1} for a total of 40, 60, 80 and 120 deposition cycles (**1tf$_{40}$**, **1tf$_{60}$**, **1tf$_{80}$** and **1tf$_{120}$**, respectively). In each deposition cycle, the functionalized QCM substrate was alternatingly exposed to the precursor solutions as follows: $Cu(OAc)_2 \cdot H_2O$ (0.5 mM in

ethanol) 10 min, ethanol (rinse) 5 min, the mixed organic linkers (H_2DE-bdc + dabco for **1tf$_x$** and H_2BME-bdc + dabco for **2tf$_x$**, 0.2 mM in ethanol) 10 min and finally ethanol 5 min. Note that, the QCM frequency change was monitored in-situ during the fabrication process. In addition, **1tf** was fabricated on the —pyridyl terminated QCM substrate by LPE process for 60 cycles (named as **1tf$_{60\text{-}Py}$**) in order to study the influence of crystallite orientation on the structural flexibility of the MOF thin-films. For comparison, the prototypic layered-pillared Cu_2(bdc)$_2$(dabco) (**3**) thin-film was prepared by LPE for 60 cycles using commercially available 1,4-benzenedicarboxylic acid (H_2bdc) as the carboxylate linker (named as **3tf$_{60}$**).

Characterisation of MOF thin-films. Surface morphology and surface coverage were investigated by a field emission scanning electron microscope (FE-SEM, ZEISS Gemini Sigma 300 VP). Cross-sectional SEM images were measured by a JEOL JSM-7500F operated in Gentle Beam mode without sputtering in order to determine the film thickness. Sorption properties of the MOF thin-films fabricated on QCM substrates were carried out on an environmental-controlled QCM (BEL-QCM-4 instrument, MicrotracBEL Corp.) at controlled temperature (25 °C) using methanol as probe molecule (the schematic setup is illustrated in Supplementary Figure 3). Prior to the sorption measurements, the films were activated *in-situ* within the BEL-QCM instrument by purging with dry He gas with a flow rate of 100 sccm for 2 h until the change of QCM frequency was stable within the range of ±5 Hz in 20 min. The mass of the MOF film was determined by conversion of the frequency difference between the MOF-deposited QCM substrate and the fundamental frequency of the SAM-functionalised QCM substrates, according to Sauerbrey's equation.[2] Afterwards, methanol sorption isotherms at 25 °C were collected by varying P/P_0 of saturated methanol vapour in He gas flow from 0.0 to 95.0%. The adsorption amounts were calculated according to the Sauerbrey's equation:

$$\Delta F = -\frac{2F_0^2}{A\sqrt{\mu \cdot \rho}} \cdot \Delta \mathrm{M} \qquad (1)$$

F_0 : fundamental frequency, A : surface area of electrode, μ : shear stress of quartz (2.947×10^{10} kg·m^{-1}·s^{-2}) and ρ : density of quartz (2648 kg·m^{-3}).

The adsorption amount at each P/P_0 (ΔM) on the MOF thin-films (M_0) was derived as:

$$\text{Adsorption amount (g/g):} \quad \frac{\Delta M}{M_0} = \frac{F - F_S}{F_S - F_0} \qquad (2)$$

M_0 : initial weight of the MOF film sample, F : measuring frequency at each relative vapour pressure and F_s : frequency after final activation of the MOF film sample.

In-situ synchrotron X-ray diffraction during methanol adsorption. Crystalline phase and structural flexibility during methanol adsorption of the MOF bulk powders and thin-films were identified by grazing incidence XRD (GIXRD, Beamline BL9 of the DELTA synchrotron radiation source, Dortmund, Germany).[3] An X-Ray energy of 15.0 keV corresponding to a wavelength of 0.827 Å was used. The angle of incidence was 0.6°). The diffraction images were converted to diffraction patterns utilizing the software package Fit2D.[4] In order to investigate structural flexibility of the MOFs upon adsorption and desorption of polar guests (herein, methanol) at room temperature (25 °C), the sample stage was connected to a temperature-controlled graphite-dome (DHS 1100, Anton Paar). This sample stage was further connected with a custom-built He gas flow system, which could semi-quantitatively control the dosed amount of methanol vapour into the sample chamber (the schematic setup is illustrated in Supplementary Figure 4). The MOF sample was placed in the sample chamber and covered with the graphite dome. The temperature of the sample chamber was kept constant at 25 °C during the whole experiment. In-situ GIXRD patterns were recorded in different stages of the methanol sorption process as follows: 1) the as-synthesised, solvated stage (mimicked by dropping methanol onto the sample); 2) the activation process (by purging the sample with He gas); 3) the methanol adsorption with 4 different relative vapour pressures (P/P_0) of 10%, 20% , 50% and 80%; and 4) the desorption (by purging with He gas). To control the P/P_0, the pressure-controlled valves at the pure He gas line and the one that passed through the methanol vaporiser were adjusted to change the mixing ratio, while keeping the total pressure at the outlet constant.

---

2. Supplementary Figures

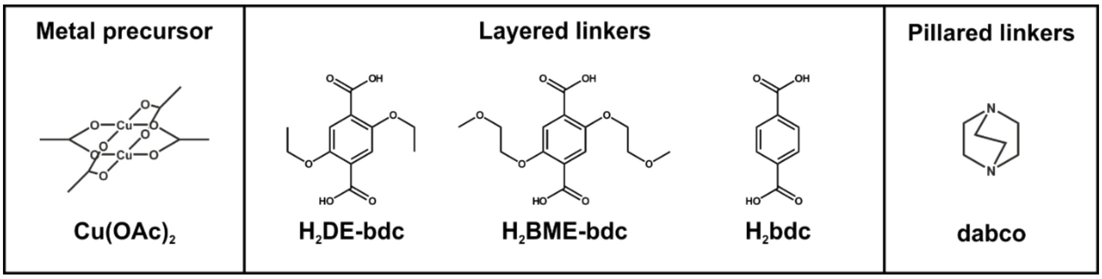

Supplementary Figure 1│ Chemical structure of metal and organic precursors used for preparation of the Cu-based layered-pillared MOFs.

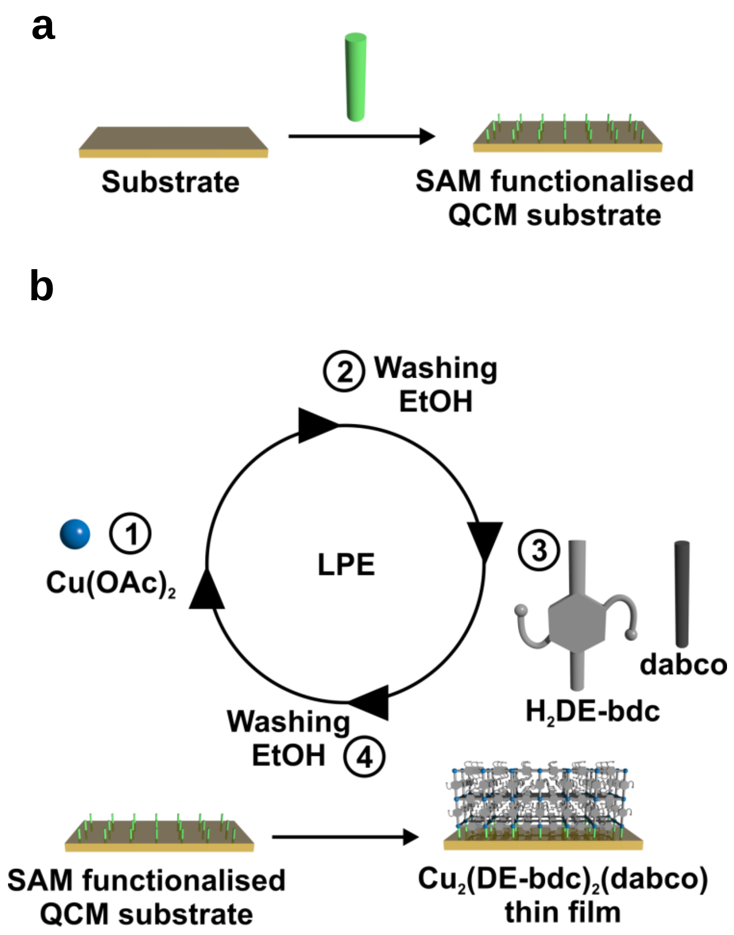

Supplementary Figure 2│ Stepwise liquid-phase epitaxial growth of Cu_2(DE-bdc)$_2$(dabco) (1tf$_x$) thin-films. (a) The Au-coated QCM substrate was firstly functionalised with the SAM providing a surface terminated with a suitable functionality (either COOH or pyridyl). (b) In each LPE deposition cycle, the functionalized QCM substrate was alternatingly exposed to the precursor solutions as follow: $Cu(OAc)_2$ (10 min), ethanol (5 min), a mixture of H_2DE-bdc and dabco (10 min), and ethanol (5 min). The fabrication was operated in the continuous flow mode at a controlled temperature of 40 °C. Note that, H_2BME-bdc and H_2bdc were used instead of H_2DE-bdc for the LPE growths of **2tf$_x$** and **3tf$_x$**, respectively.

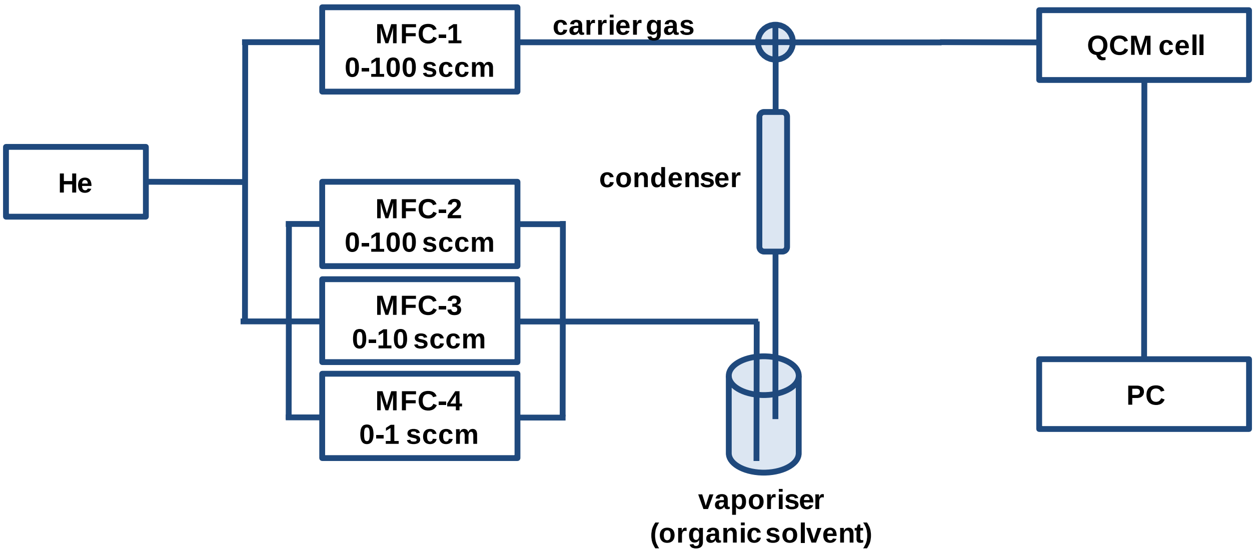

Supplementary Figure 3 | Schematic experimental setup of an environmentally-controlled QCM used for organic vapour adsorption in MOF thin-films.

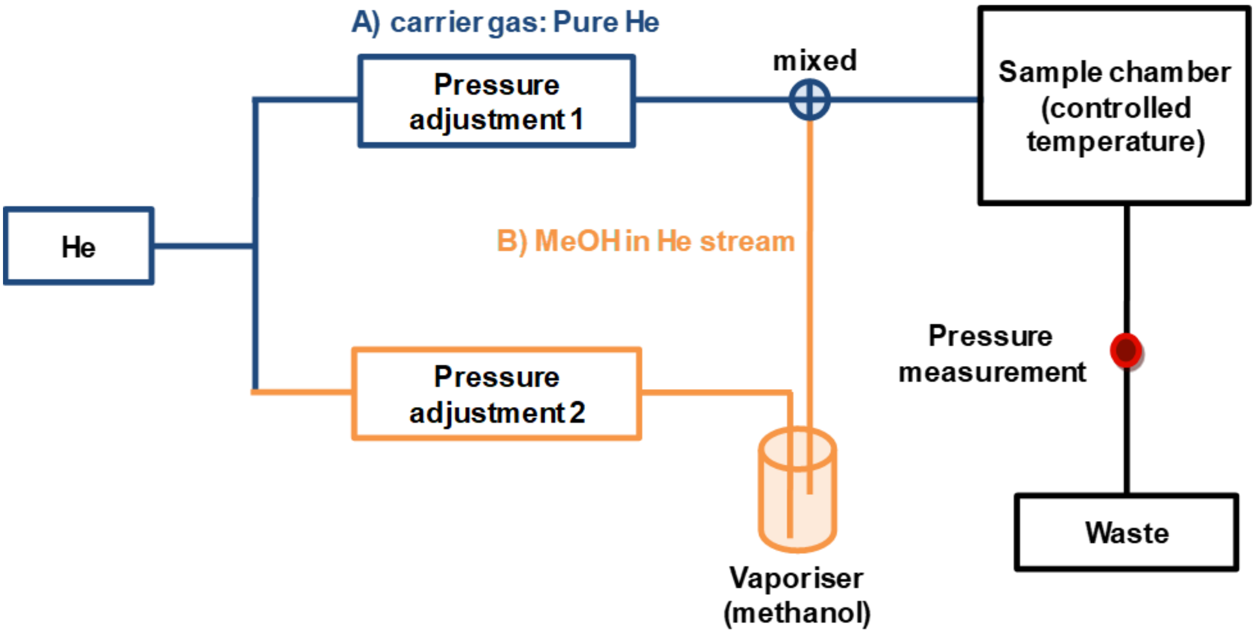

Supplementary Figure 4 | Schematic experimental setup of the in-situ GIXRD measurements during methanol vapour adsorption.

Bulk MOF powders

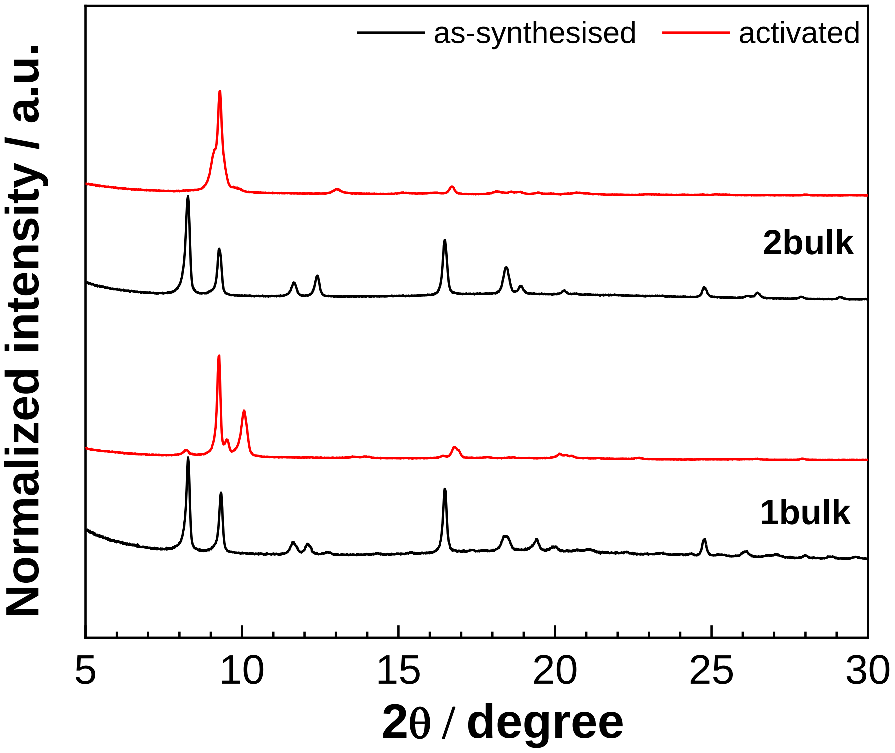

Supplementary Figure 5│ XRD patterns of as-synthesised (black) and activated (red) of 1bulk and 2bulk. (using Cu Kα radiation as an X-ray source)

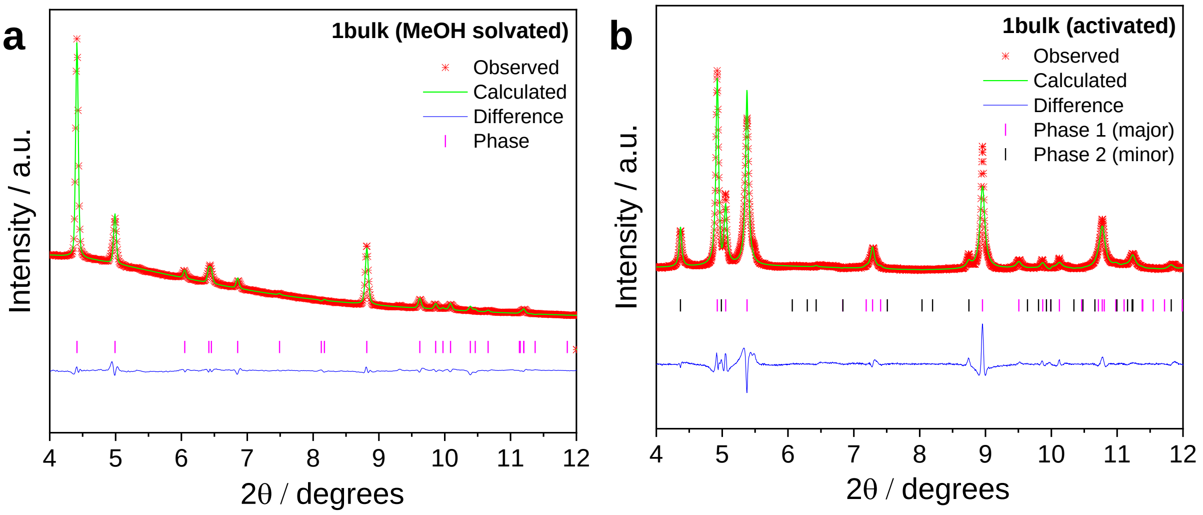

Supplementary Figure 6│ Pawley refinements of GIXRD patterns of 1bulk in (a) MeOH solvated and (b) activated form. (using synchrotron X-ray wavelength of 0.827 Å)

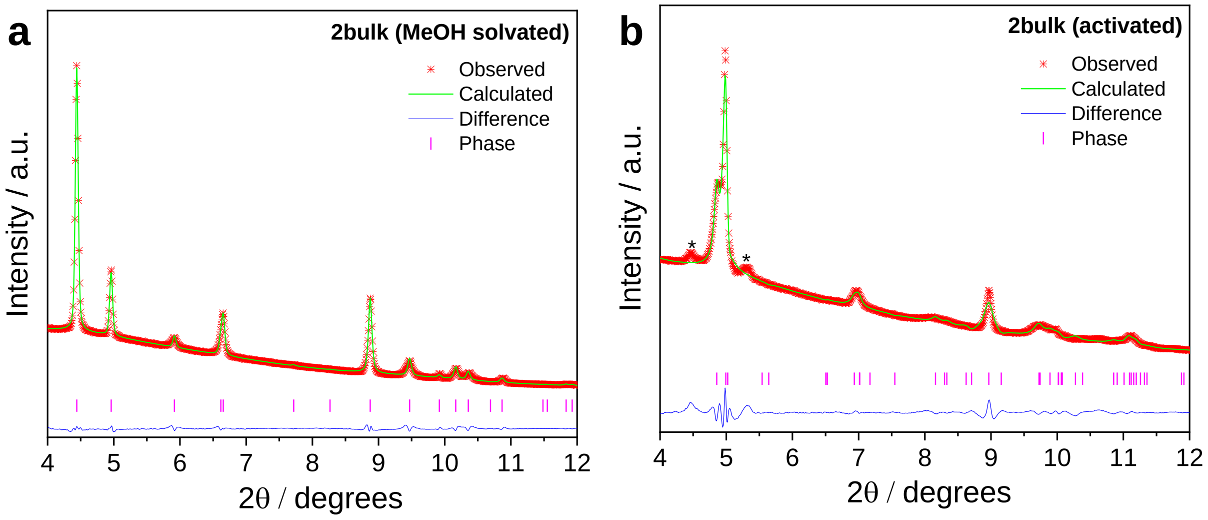

Supplementary Figure 7 | Pawley refinements of GIXRD patterns of 2bulk in (a) MeOH solvated and (b) activated form. (using synchrotron X-ray wavelength of 0.827 Å)

Supplementary Table 1 | Summary of refined lattice parameters of 1bulk and 2bulk samples in MeOH-solvated and activated form based on Pawley refinement of synchrotron-based GIXRD data.

**Refined lattice parameters**	**1bulk**			**2bulk**	
	**MeOH solvated**	**activated**		**MeOH solvated**	**activated**
**Space group**	C 2/m	C 2/m	C 2/m	C mmm	P 21/m
**a (Å)**	15.779760	18.772260	15.67380	16.06264	18.82834
**b (Å)**	14.816613	9.972752	15.05001	14.36069	11.37029
**c (Å)**	9.568058	9.626891	9.525823	9.580806	9.468338
**α (°)**	90	90	90	90	90
**β (°)**	95.0695	93.6579	95.0979	90	91.2751
**γ (°)**	90	90	90	90	90
**Volume (Å^3)**	2228.286	1798.589	2238.166	2210.009	2026.515
**Notation**	**1bulk** solvated, **lp** phase	**1bulk** activated, **np** phase	**1bulk**, **lp** phase #	**2bulk** solvated, **lp** phase	**2bulk** activated, **np** phase

Note: The **1bulk**, **lp** phase appears as a minor phase in **1bulk** activated form, which may occur due to the incomplete activation process of the MOF powder sample.

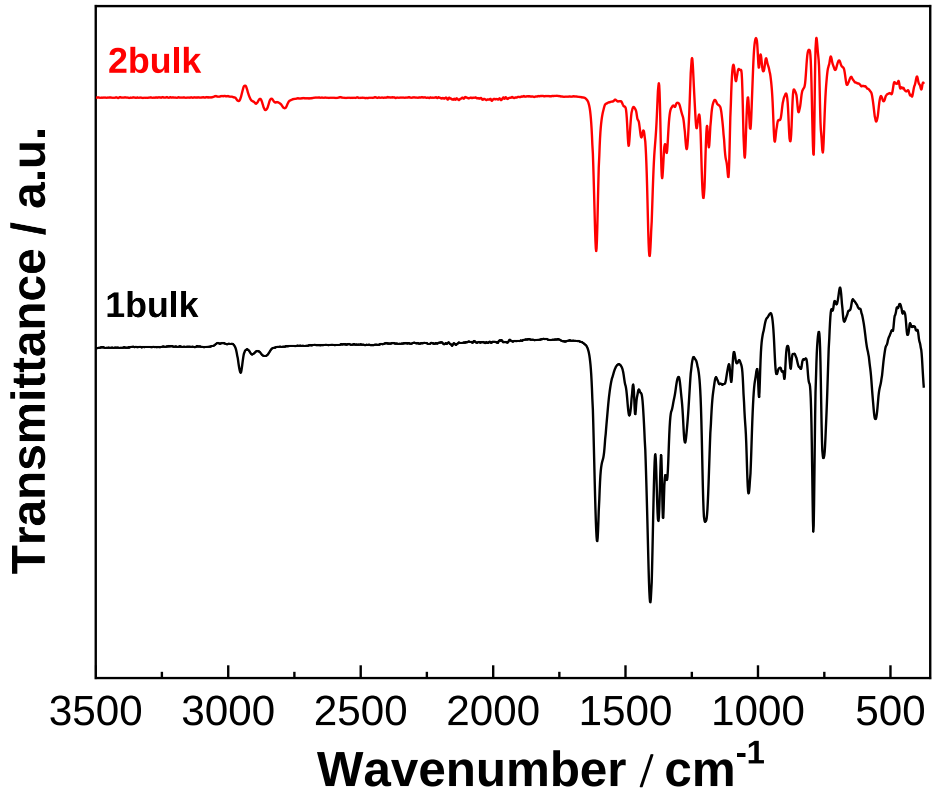

Supplementary Figure 8│ FT-IR spectra of activated 1bulk and 2bulk.

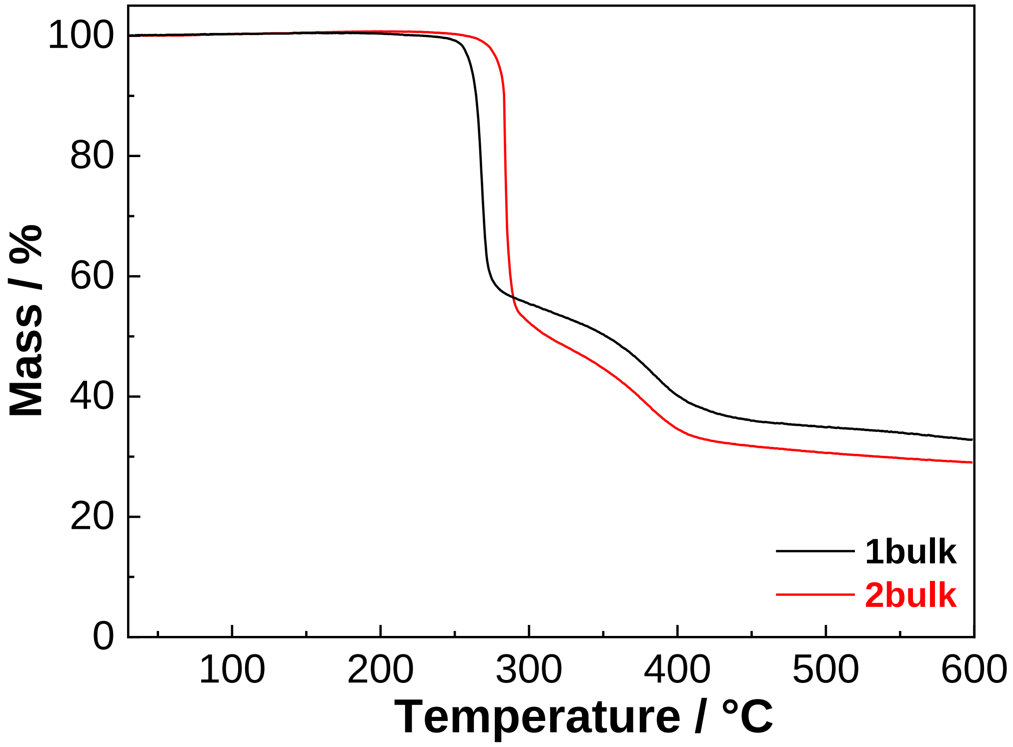

Supplementary Figure 9│ TG traces of activated 1bulk and 2bulk.

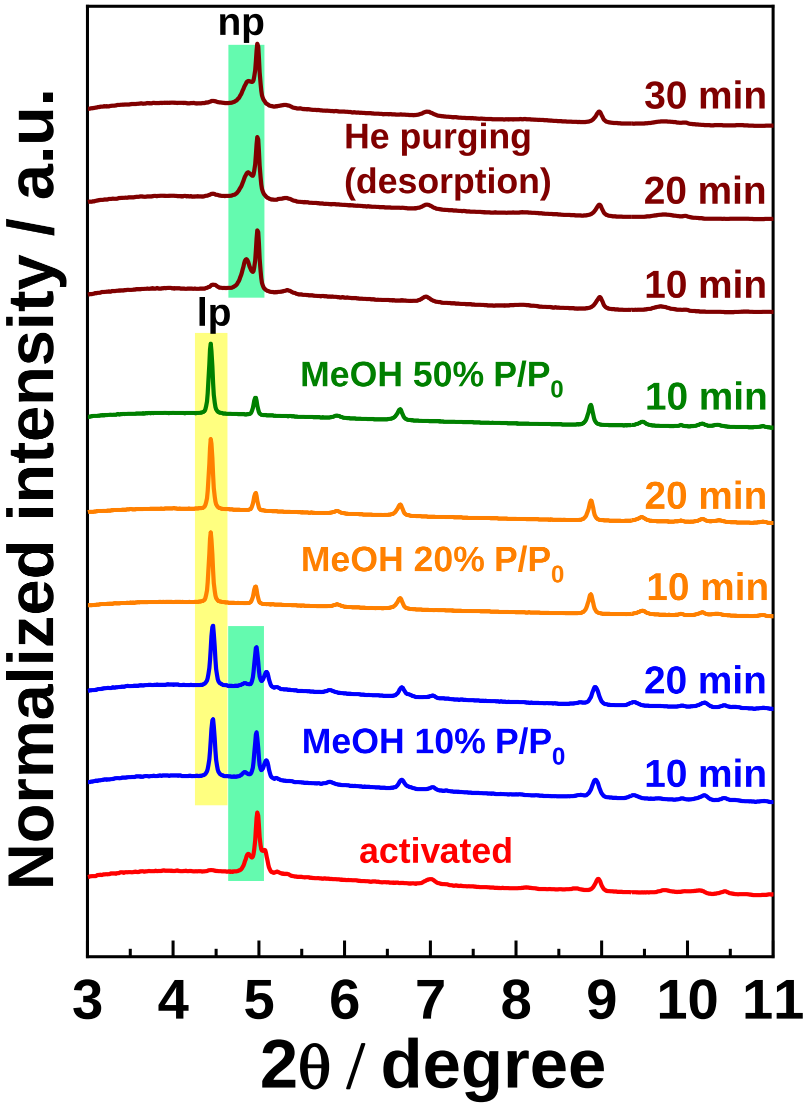

Supplementary Figure 10│ GIXRD profiles of 2bulk upon methanol sorption at 25 °C. (using synchrotron X-ray wavelength of 0.827 Å). Adsorption of 10% P/P_0 methanol initiates the framework breathing transition from the activated, narrow-pore (**np**) form to the solvated, large-pore (**lp**) form. **2bulk** is fully converted to the solvated, **lp** form after adsorption of methanol from 20% P/P_0 and higher. Purging the **2bulk** sample with He gas at 25 °C desorbs methanol out of the framework and shows the reversible structural transition of **2bulk** back to the activated, **np** phase. Note that, the representative diffraction peaks of the **lp** and **np** phase are highlighted in yellow and green, respectively.

$Cu_2(DE\text{-}bdc)_2(dabco)$ thin-films ($1tf_x$)

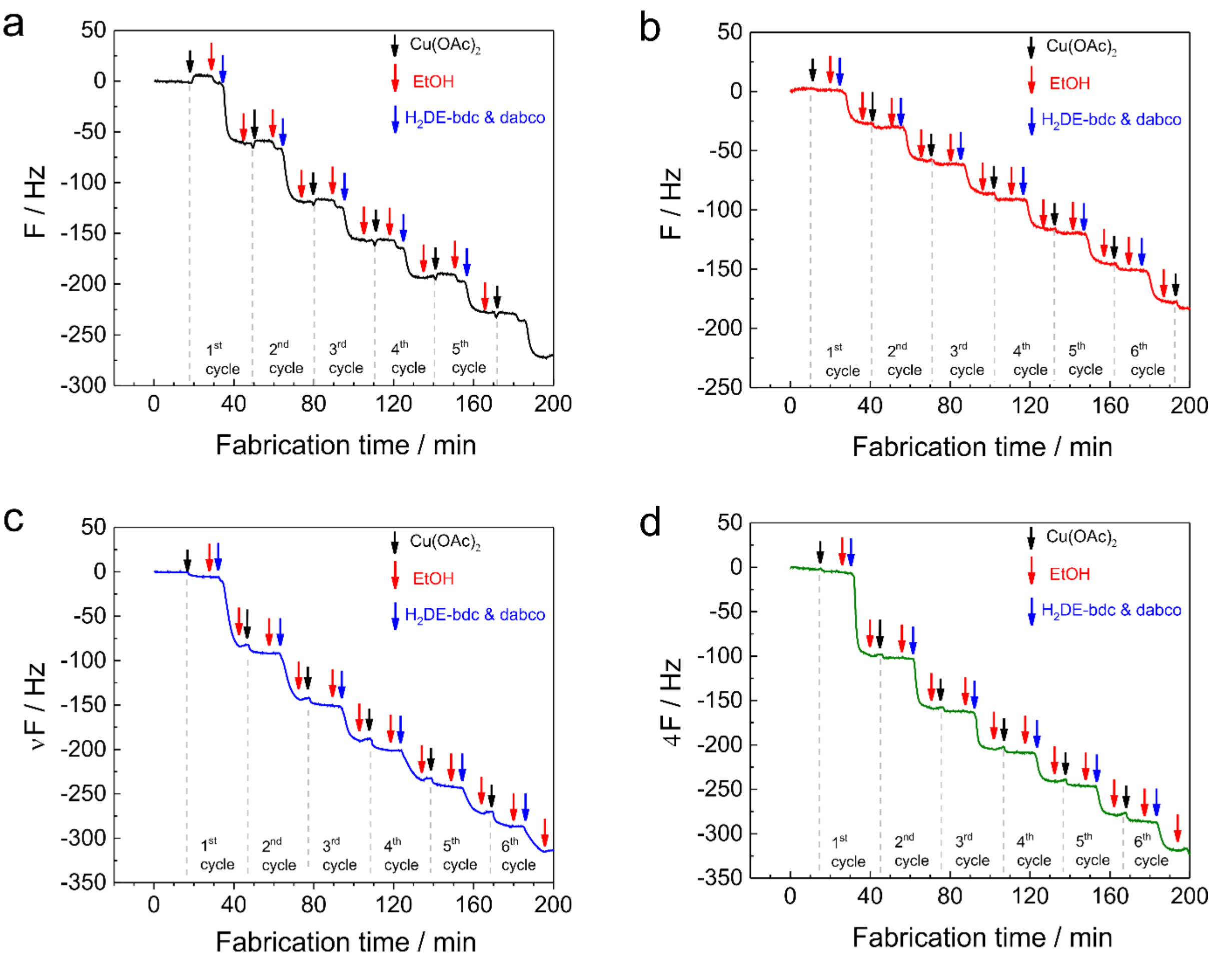

Supplementary Figure 11│ Changes in the QCM oscillator frequency (*F*) as a function of time at the early stage of the LPE growth of (a) $1tf_{40}$, (b) $1tf_{60}$, (c) $1tf_{80}$, and (d) $1tf_{120}$. Generally, the LPE growth of **$1tf_x$** shows saturation during each deposition step of the metal and the organic linkers, indicating a self-terminated growth of the layered-pillared MOF thin-films by the LPE process.

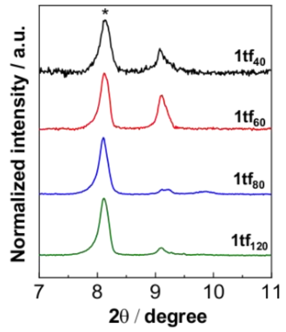

Thin films	2θ of the peak (°)	FWHM (°)	Crystallite size (nm)
**1tf40**	8.13374 ± 0.00319	0.21496 ± 0.00769	37.069
**1tf60**	8.14935 ± 0.00130	0.20939 ± 0.00323	38.055
**1tf80**	8.10086 ± 0.00108	0.19916 ± 0.00260	40.009
**1tf120**	8.11160 ± 0.00119	0.19731 ± 0.00285	40.384

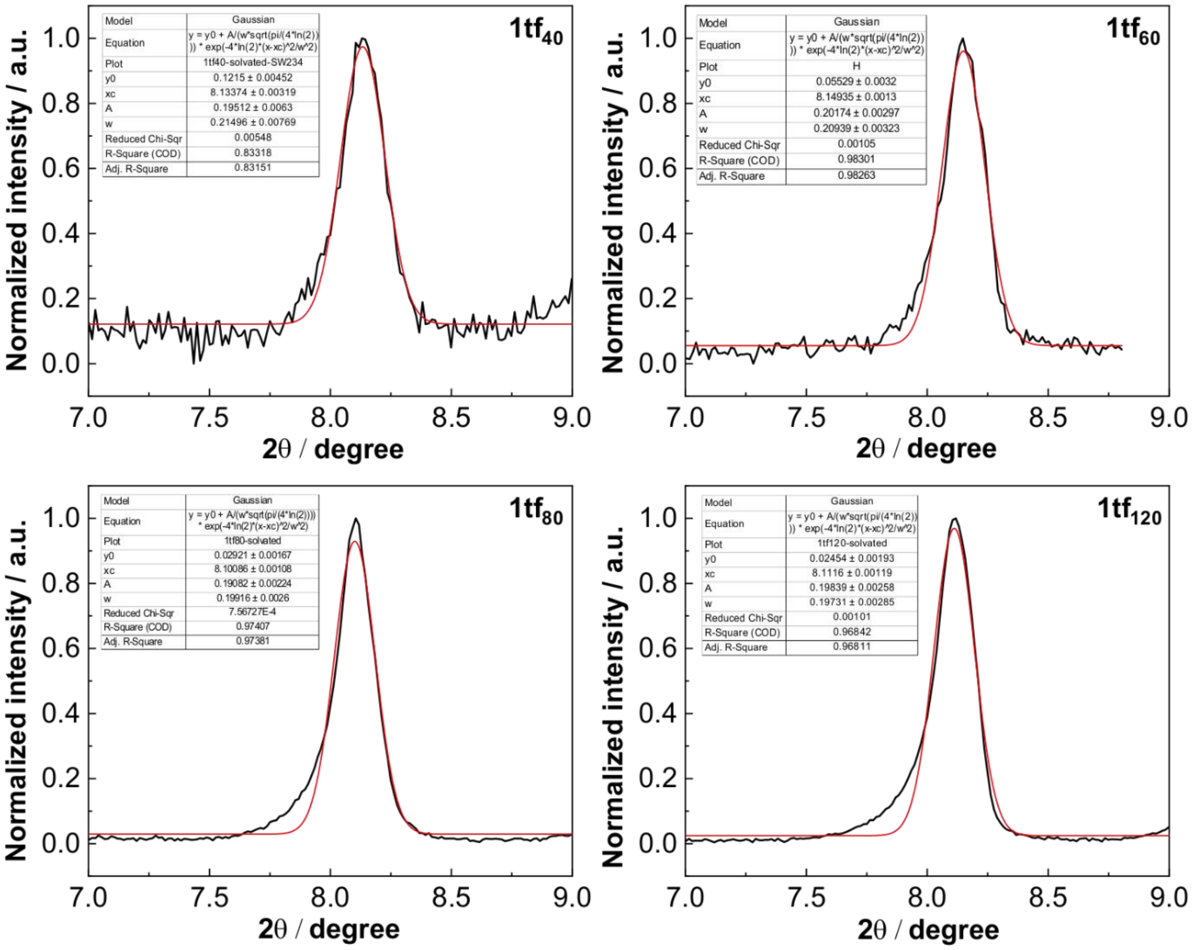

Supplementary Figure 12│ Crystallite size of MeOH-solvated 1tf$_x$ samples calculated from GIXRD patterns by using Scherrer Equation. (using Cu Kα radiation as an X-ray source). Prior to the GIXRD measurement, MeOH (1 drop) is added on the **1tf$_x$** films for preparing the solvated form. GIXRD measurement was measured shortly after adding MeOH to the thin-film sample using 2θ-scan in the range of 7° to 11°, which is covered the main diffraction peaks of the MOF **1**.

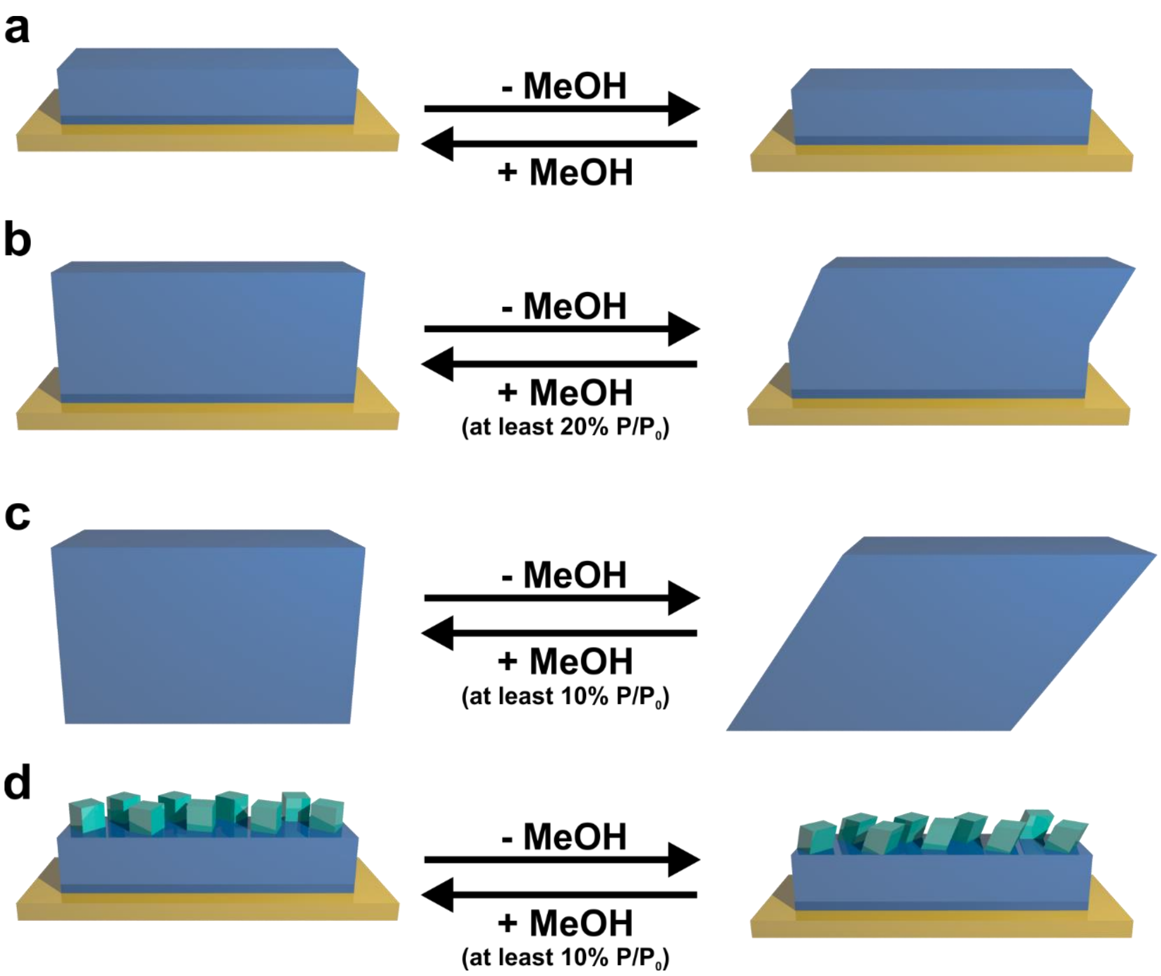

Supplementary Figure 13│ Schematic representation of the possibilities for structural responsiveness upon methanol sorption process of the MOF 1. (a) **1tf**$_{40}$ exhibits a restricted framework flexibility due to strong interactions of the MOF crystallites with the substrate surface leading to a high activation barrier for the **lp**-to-**np** transformation. Hence, only the **lp** form is observed, regardless of the sample treatment; (b) The larger crystallite-dimension films (**1tf**$_{60}$, **1tf**$_{80}$, or **1tf**$_{120}$) exhibit framework flexibility with some restrictions. Only the crystallite domains at the top, further away from the surface can undergo the transition between **np** and **lp** forms during methanol adsorption, while the bottom part which is strongly attached to the surface still remains in the **lp** form. MeOH of at least 20% P/P_0 is required to initiate the framework transformation; (c) **1bulk** undergoes a breathing transformation upon methanol sorption already at 10% P/P_0; (d) If the obtained materials consisting of the low crystallite-dimension thin film mixed with the bulk powder on top of it, the bulk powder should undergo the breathing phenomenon by introduction of methanol also at low P/P_0 MeOH i.e. at least 10%. Moreover, all of the "loose" bulk crystals on top of the thin film would have been easily flushed away during the washing step with ethanol in the LPE process. Note that, the schematic representation shown herein is only a suggested structure based on lattice refinements and is not a depiction of the actual structure.

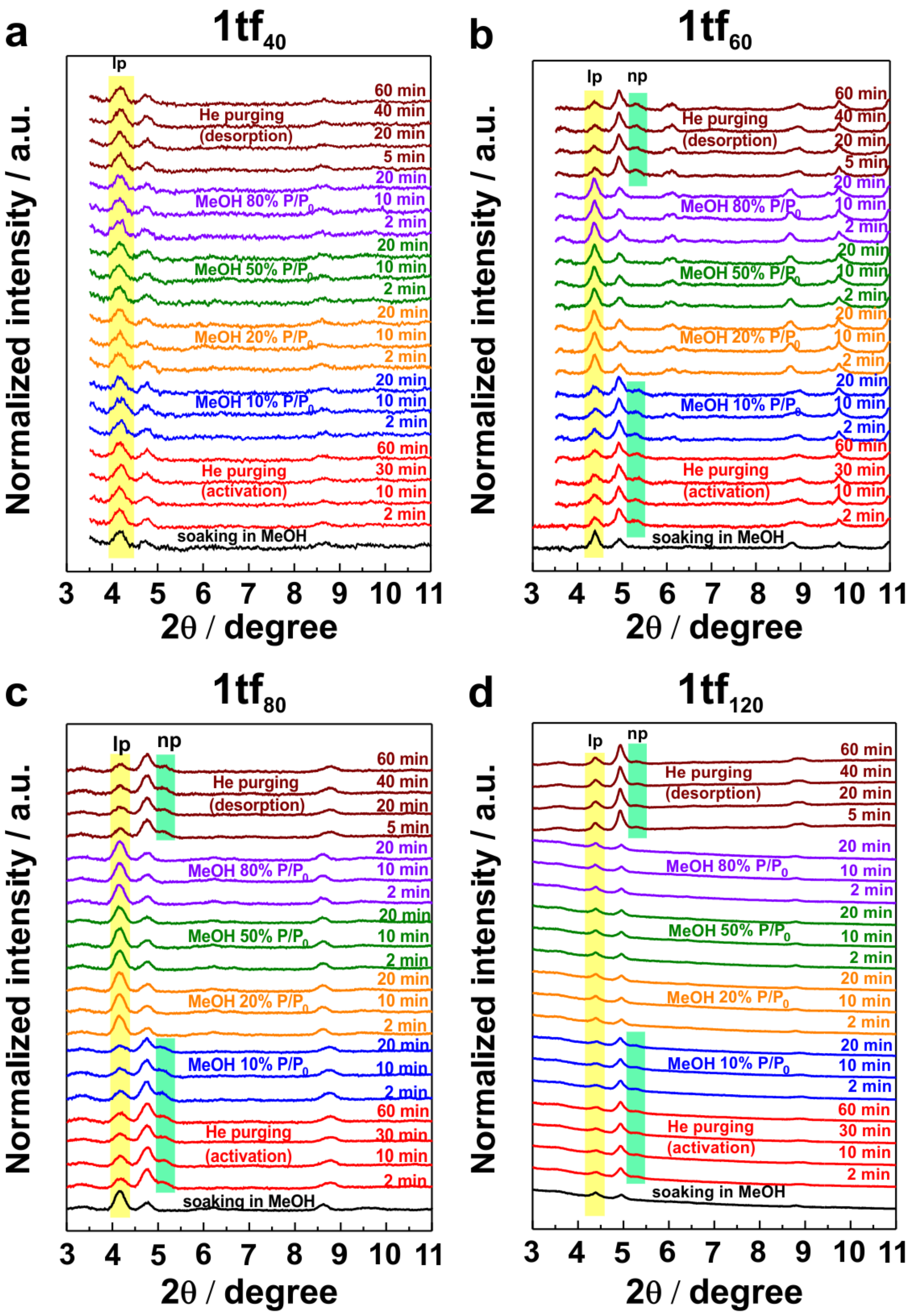

Supplementary Figure 14 │ In-plane GIXRD profiles of the 1tf$_x$ materials during in-situ methanol sorption at 25 °C. (a) **1tf$_{40}$**, (b) **1tf$_{60}$**, (c) **1tf$_{80}$** and (d) **1tf$_{120}$** were fabricated on the —COOH functionalised QCM substrates. The **1tf$_x$** materials exhibit a significantly different structural flexibility upon methanol sorption than **1bulk**. A marked dependence of the structural flexibility on the total number of fabrication cycles is observed. Note that, the highlighted labels in yellow and green color represent the main diffraction peak belonging only to the **lp** and **np** phase, respectively.

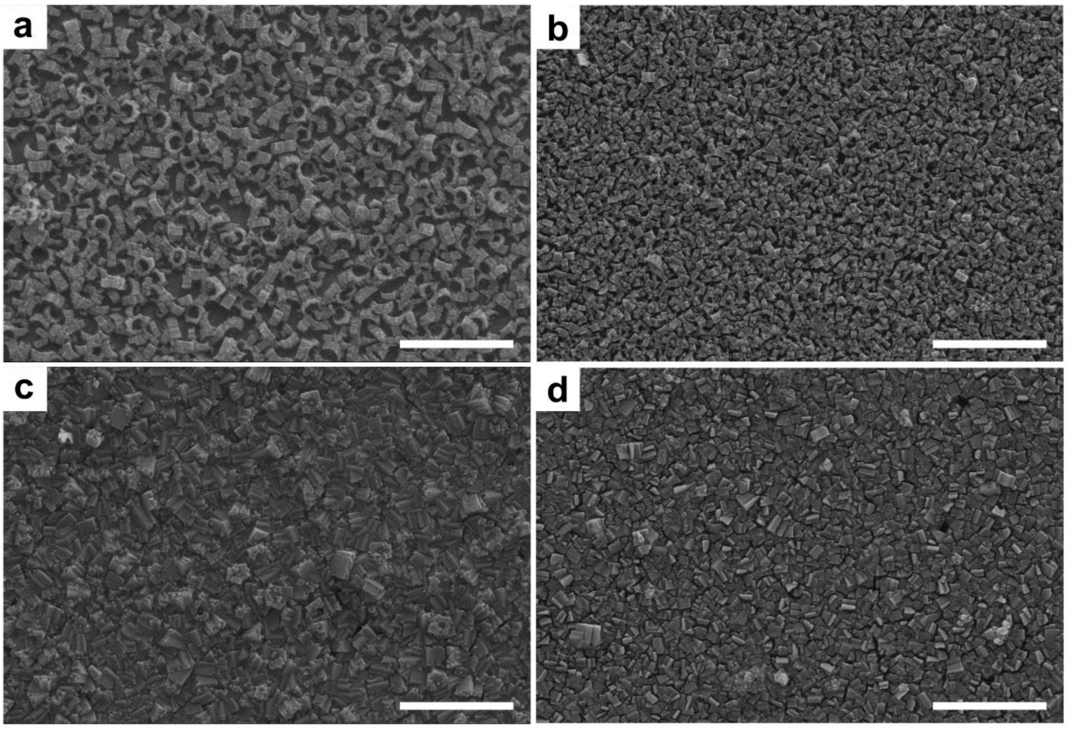

Supplementary Figure 15│ Top-view SEM images of the thin films: (a) **1tf$_{40}$**, (b) **1tf$_{60}$**, (c) **1tf$_{80}$**, and (d) **1tf$_{120}$** fabricated by LPE process on the —COOH functionalised QCM substrates. Scale bar at each panel represents a length of 5 μm.

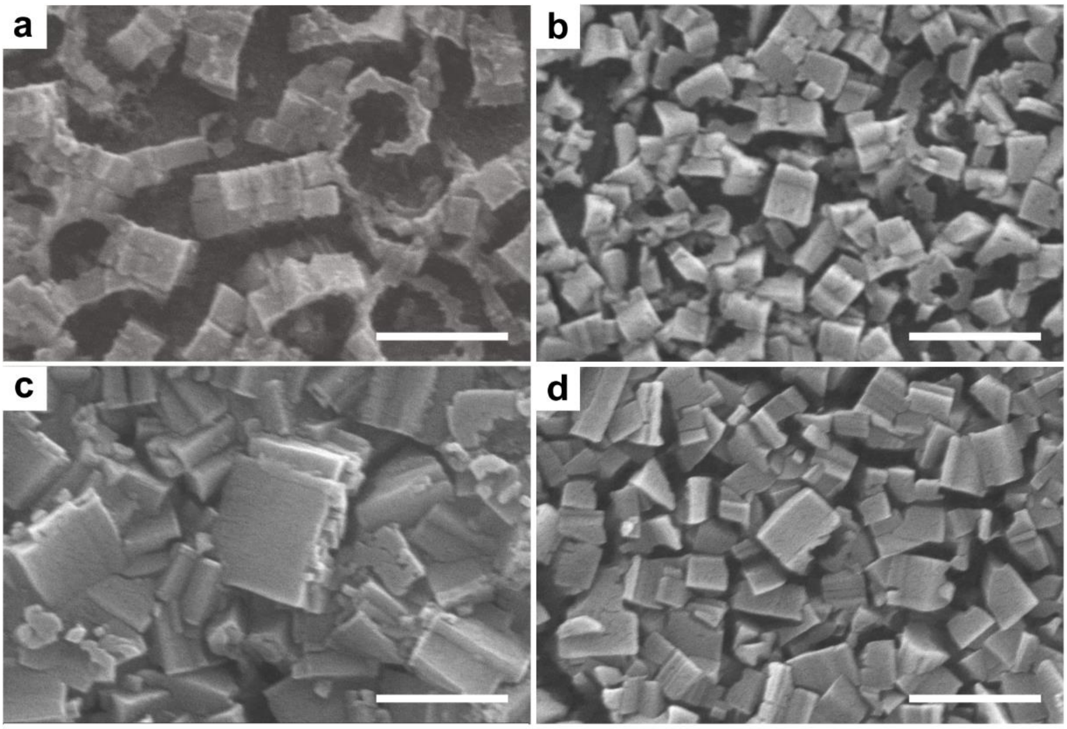

Supplementary Figure 16│High-magnification SEM images of the thin films: (a) **1tf$_{40}$**, (b) **1tf$_{60}$**, (c) **1tf$_{80}$**, and (d) **1tf$_{120}$** fabricated by LPE process on the —COOH functionalised QCM substrates. Scale bar at each panel represents a length of 1 μm.

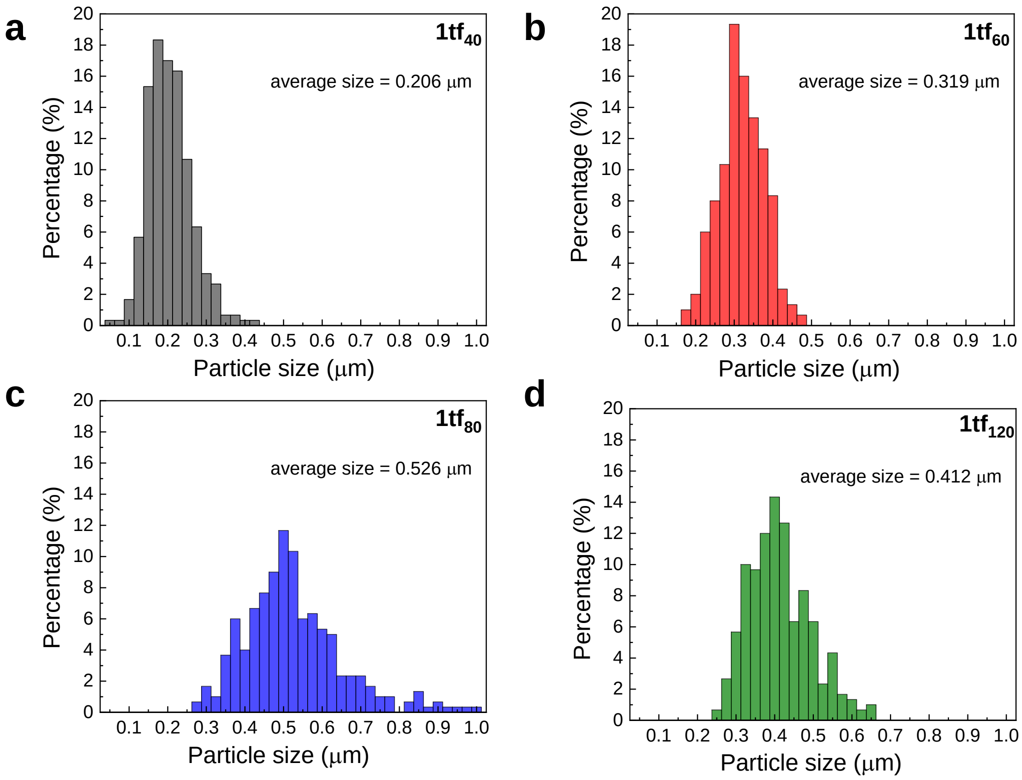

Supplementary Figure 17│ Particle size distribution measured from the SEM images of the thin films: (a) **1tf$_{40}$**, (b) **1tf$_{60}$**, (c) **1tf$_{80}$**, and (d) **1tf$_{120}$** fabricated by LPE process on the —COOH functionalised QCM substrates.

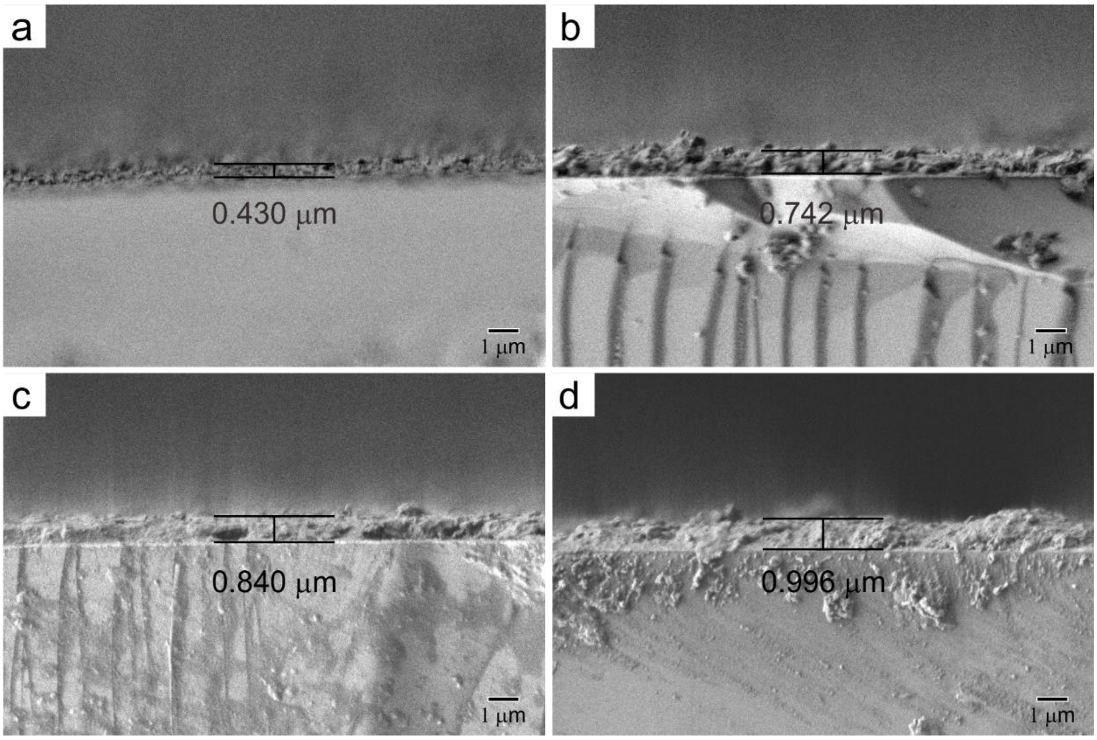

Supplementary Figure 18│ Film thickness of (a) 1tf$_{40}$, (b) 1tf$_{60}$, (c) 1tf$_{80}$, and (d) 1tf$_{120}$ measured from the cross-sectional SEM images. The thin films were fabricated by LPE process on the —COOH functionalised QCM substrates.

Supplementary Table 2 │ Comparison of crystallite size (calculated from GIXRD based on Scherrer Equation), average particle size (top-view SEM images), and film thickness (cross-sectional SEM images) of the 1tf$_x$ samples.

Thin-film samples	Crystallite size (nm)	Particle size (µm)	Film thickness (µm)
**1tf$_{40}$**	37.069	0.206	0.430
**1tf$_{60}$**	38.055	0.319	0.742
**1tf$_{80}$**	40.009	0.526	0.840
**1tf$_{120}$**	40.384	0.412	0.996

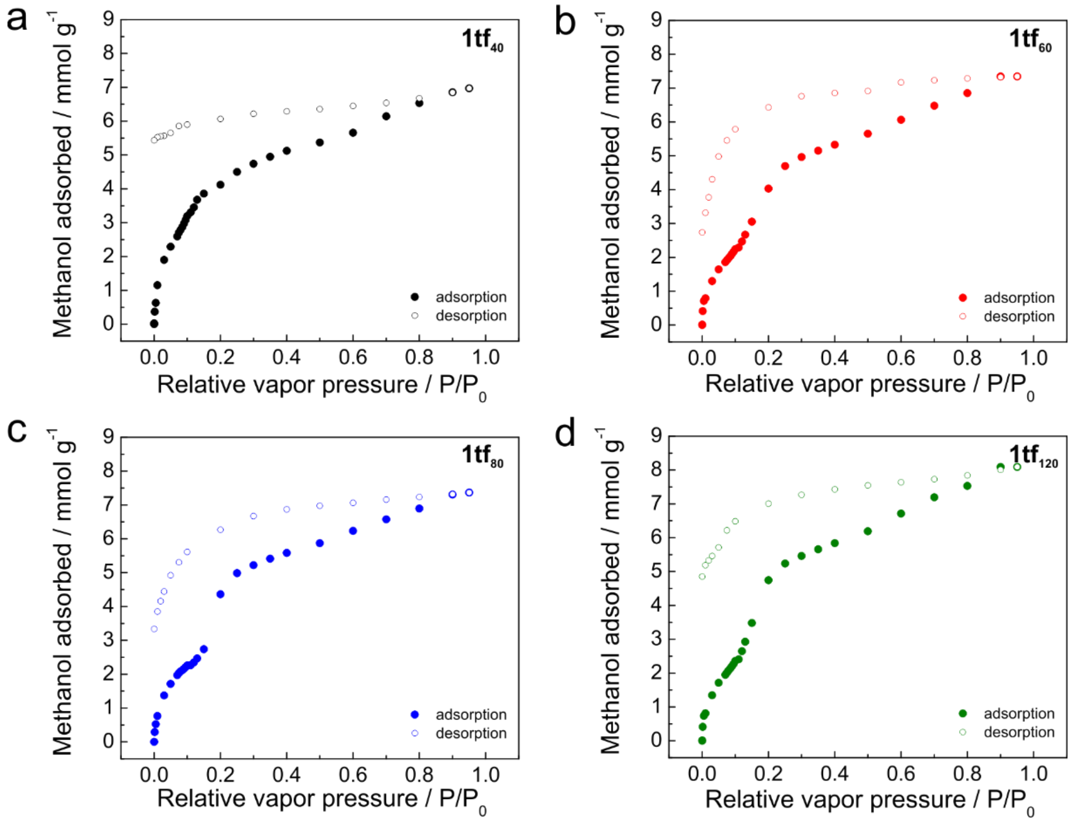

Supplementary Figure 19│Methanol adsorption isotherms at 25 °C of (a) 1tf$_{40}$, (b) 1tf$_{60}$, (c) 1tf$_{80}$, and (d) 1tf$_{120}$ measured by environmentally-controlled QCM. Filled and empty symbols depict adsorption and desorption. **1tf$_{40}$** exhibits a single-step methanol adsorption, whereas **1tf$_{60}$**, **1tf$_{80}$** and **1tf$_{120}$** show two-steps methanol adsorption. A significant change in the slope of the methanol adsorption isotherm at P/P_0 of 0.15 indicates the starting point of the framework transformation from **np** to **lp**. In correspondence with the in-situ GIXRD profiles during the methanol adsorption process, these methanol adsorption data reveal the dependency of the structural flexibility of **1tf$_x$** on the total number of LPE fabrication cycles or in other word, the MOF crystallite dimension anchored at the substrate surface.

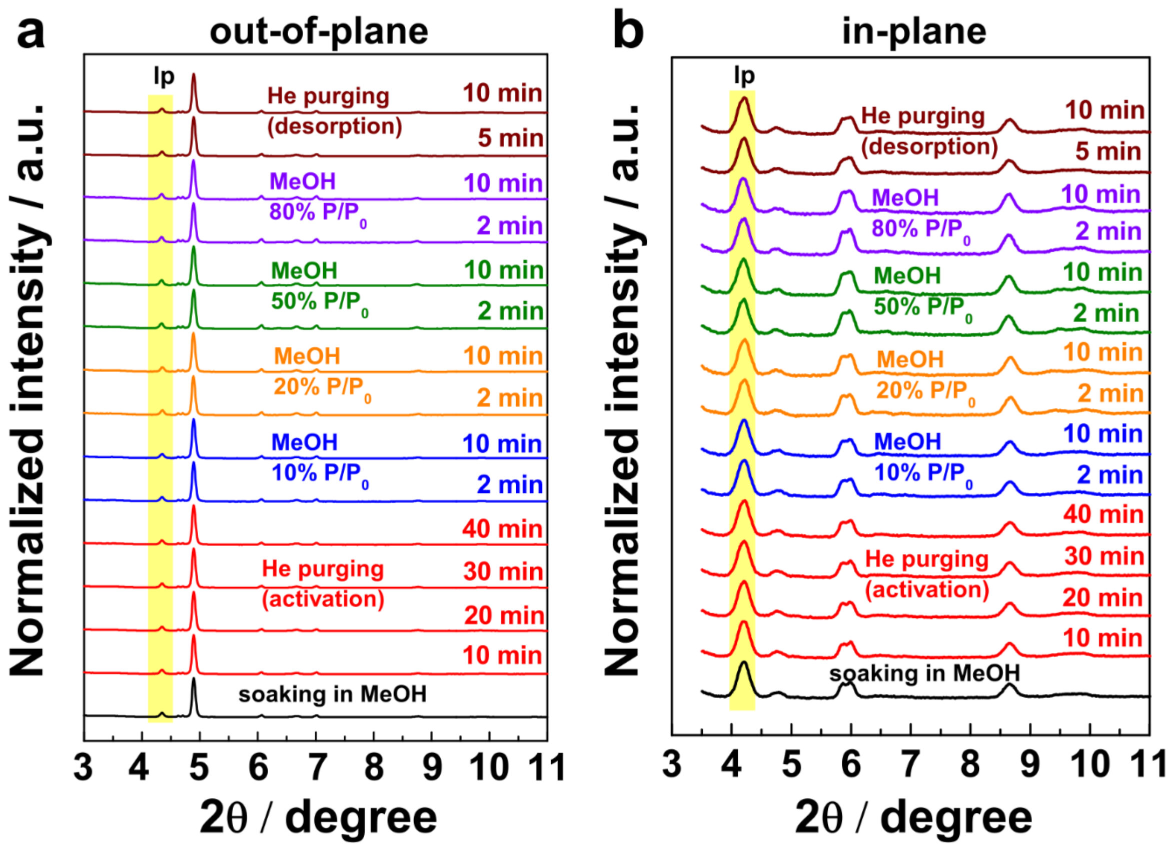

Supplementary Figure 20│ Out-of-plane and in-plane GIXRD profiles measured during methanol sorption at 25 °C of 1tf$_{60\text{-Py}}$. 1tf$_{60\text{-Py}}$ was fabricated by LPE process on the —pyridyl functionalised QCM substrates for 60 cycles. According to the out-of-plane GIXRD patterns, the as-synthesised (or MeOH-solvated) **1tf$_{60\text{-Py}}$** exhibits a preferred growth along the dabco-related orientation of the framework, which can be assigned to the (001)-orientation by referring to the previously-reported Zn-based analogous structure.[1] Hence, most of the crystal planes containing the Cu_2(DE-bdc)$_2$ grids are fixed parallelly to the substrate surface. During all methanol adsorption measurements, **1tf$_{60\text{-Py}}$** remains in the **lp** form, indicating a restriction of the framework flexibility compared to **1tf$_{60}$** prepared with an identical number of LPE cycles. This observation shows a strong dependency of the guest-induced structural flexibility of the thin-films on the orientation of the crystallites anchored to the substrate surface.

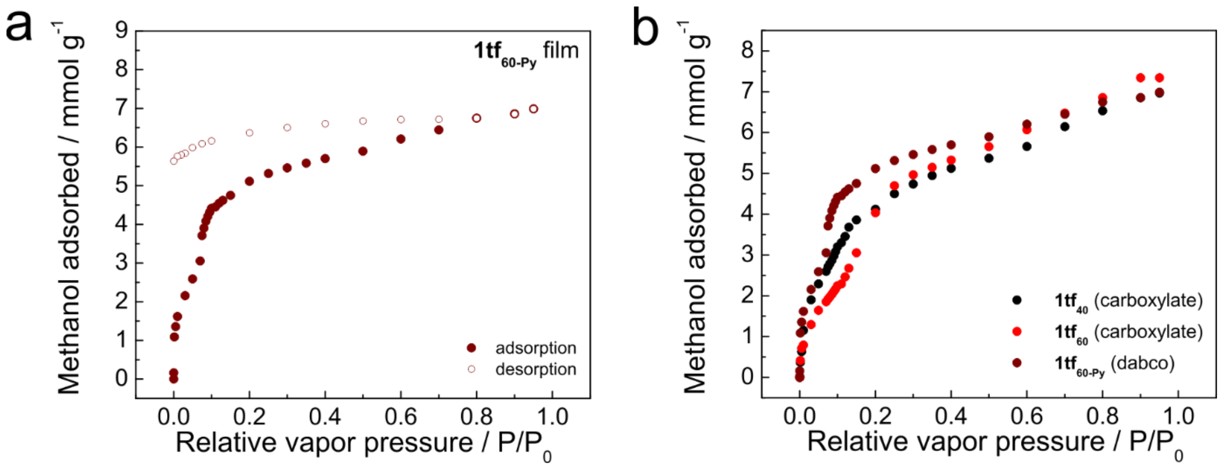

Supplementary Figure 21 | Methanol sorption isotherm at 25 °C of 1tf$_{60\text{-}Py}$. 1tf$_{60\text{-}Py}$ was fabricated by 60 LPE cycles on a —pyridyl functionalised QCM substrates. (a) **1tf$_{60\text{-}Py}$** shows preferred growth orientation along the (001) direction (referring to the previously-reported Zn-based analogous structure)[1] and shows a tiny step at P/P_0 of 0.15 in the methanol adsorption curve. However, there is no phase transition observed in the in-situ GIXRD measurement upon methanol adsorption (see also Supplementary Figure 15); (b) Comparison of the methanol adsorption profile of **1tf$_{60\text{-}Py}$** with **1tf$_{40}$** and **1tf$_{60}$**. The adsorption isotherm of **1tf$_{60\text{-}Py}$** shows a shape closer to **1tf$_{40}$** than **1tf$_{60}$**, underlining the rigid nature of the film further. In contrast, **1tf$_{60}$** film shows a two-steps adsorption profile with a change starting at P/P_0 of 0.15 due to the **np**-to-**lp** framework transformation of the crystallite domains at the upper layers of the thin film. In other words, the flexibility is inhibited if the growth direction of **1tf** is changed from along the (110) direction to the (001) direction. Note that, the total specific adsorption capacity is similar for **1tf$_{40}$**, **1tf$_{60}$** and **1tf$_{60\text{-}Py}$** when reaching high relative pressures, as all MOF crystallites are in the **lp** form at this stage, highlighting the similar quality and porosity of the obtained **1tf$_x$** samples.

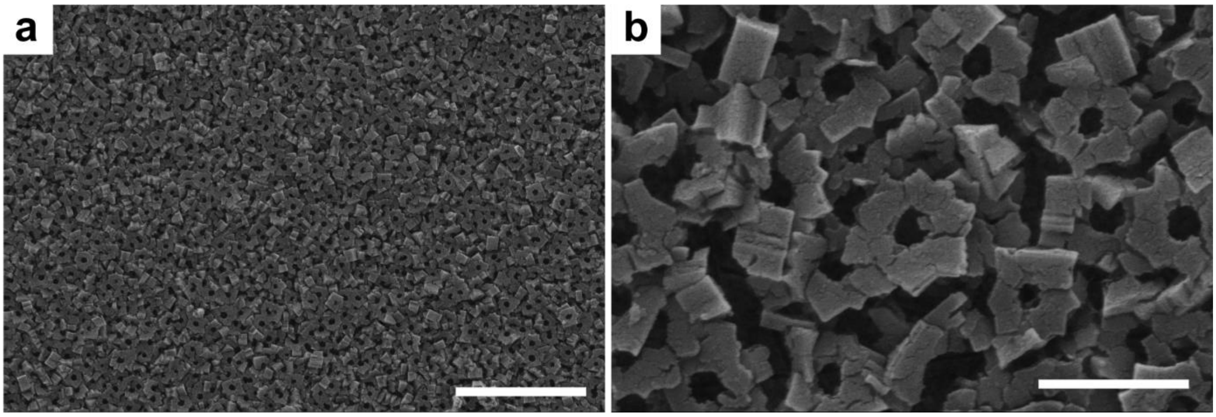

Supplementary Figure 22 | Top-view SEM micrographs of 1tf$_{60\text{-}Py}$. The film was fabricated by LPE process on the —pyridyl functionalised QCM substrates for 60 cycles. Scale bar at each panel represents a length of (a) 5 μm and (b) 1 μm.

Cu$_2$(BME-bdc)$_2$(dabco) thin-films (2tf$_x$)

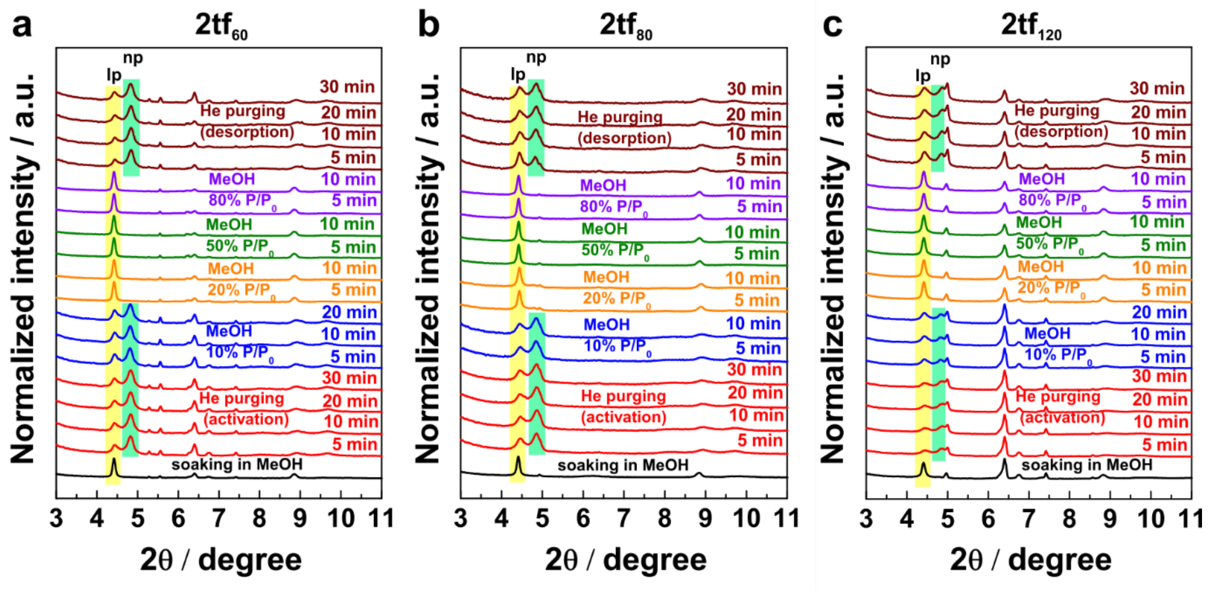

Supplementary Figure 23│ Out-of-plane GIXRD profiles of 2tf$_x$ upon in-situ methanol sorption at 25 °C. (a) **2tf$_{60}$**, (b) **2tf$_{80}$**, and (c) **1tf$_{120}$** were fabricated by the LPE process on —COOH functionalised QCM substrates. These **2tf$_x$** samples exhibit preferred growth along the (010) lattice plane (referring to the literature)[5], similar to the **1tf$_x$** analogues**.** Moreover, **2tf$_x$** also reveals a different structural responsiveness upon methanol sorption from the **2bulk.** The structural flexibility depends on the total number of LPE fabrication cycles as in the case of **1tf$_x$** films. However, the change in lattice parameters during the **np**-to-**lp** breathing transition of **2tf$_x$** is smaller than for **1tf$_x$** (see also the Pawley refinements of **1bulk** and **2bulk** in Supplementary Figure 6 and 7), highlighting the fine tuning of the guest-induced structural flexibility by the choices of alkoxy-functionalised pendent groups within the frameworks.

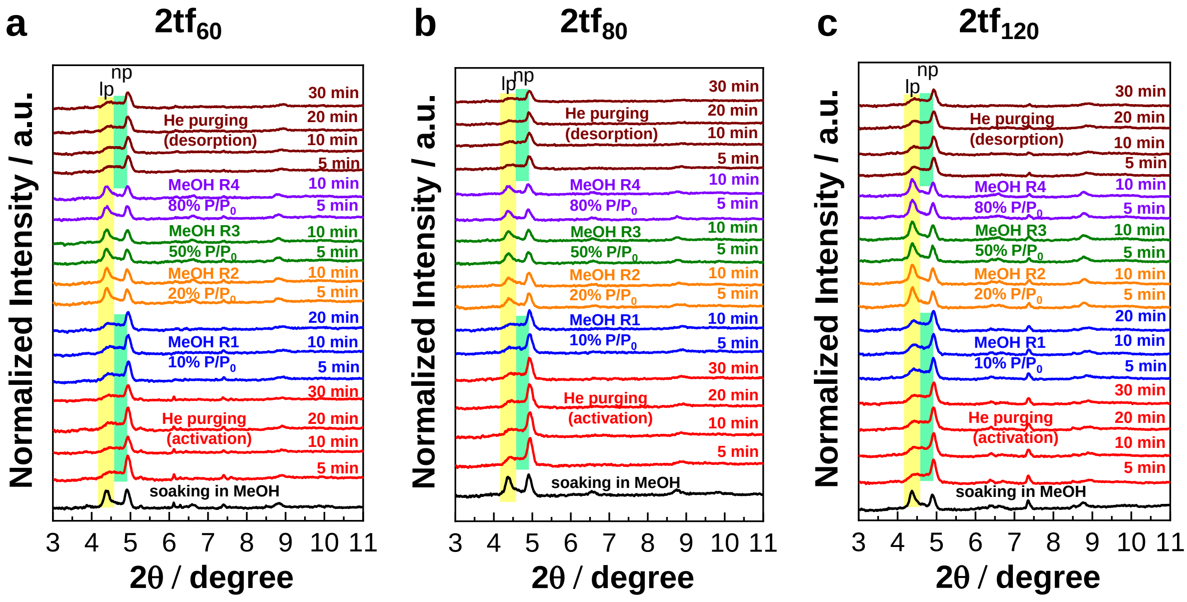

Supplementary Figure 24│ In-plane GIXRD profiles of 2tf$_x$ upon in-situ methanol sorption at 25 °C. (a) **2tf$_{60}$**, (b) **2tf$_{80}$**, and (c) **1tf$_{120}$** were fabricated by the LPE process on —COOH functionalised QCM substrates. **2tf$_x$** reveals a different structural responsiveness upon methanol sorption from the **2bulk**, and shows a dependency of the structural flexibility on the total number of LPE fabrication cycles as similar as observed in the **1tf$_x$** films. Note that, the structural flexibility of the thin-films is less clearly observed in the in-plane direction in comparison with the out-of-plane direction due to the preferred crystallite orientation of the LPE-fabricated **2tf$_x$** thin-films along the growth direction (parallel to the substrate surface).

Cu$_2$(bdc)$_2$(dabco) thin-films (3tf$_x$)

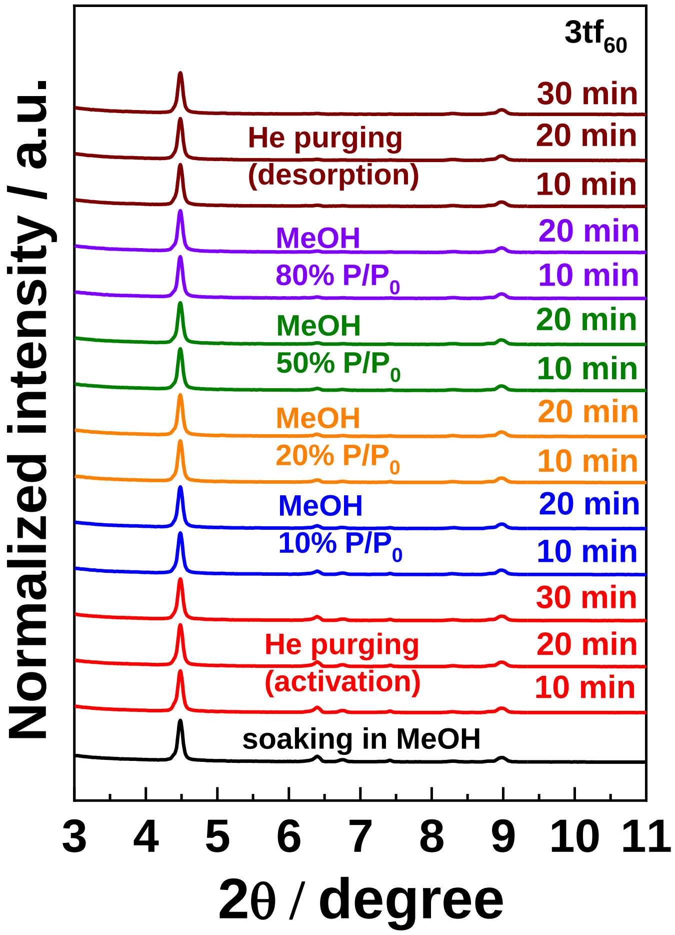

Supplementary Figure 25 | Out-of-plane GIXRD profiles of the 3tf$_{60}$ upon in-situ methanol sorption at 25 °C. The **3tf$_{60}$** was fabricated by the LPE process on the —COOH functionalised QCM substrates (60 cycles). No structural transition is observed during the methanol adsorption process and hence the MOF framework structure remains unchanged. This result emphasizes that the framework flexibility of the Cu-based layered-pillared MOFs thin films is initiated by the introduction of the alkoxy-functionalised pendent sidechains at the benzene-1,4-dicarboxylate linkers.

3. Supplementary References

1. Henke S., Schneemann A., Wütscher A. & Fischer R. A. Directing the breathing behavior of pillared-layered metal-organic frameworks via a systematic library of functionalized linkers bearing flexible substituents. *J. Am. Chem. Soc.* **134**, 9464-9474 (2012).
2. Sauerbrey G. Verwendung von Schwingquarzen zur Wägung dünner Schichten und zur Mikrowägung. *Z. Phys.* **155**, 206-222 (1959).
3. Krywka, C. *et al*. The small-angle and wide-angle X-Ray scattering set-up at beamline BL9 of DELTA. *J. Synchrotron Radiat.* **14**, 244-251 (2007).
4. Hammersley, A. P., Svensson, S. O., Hanfland, M., Fitch, A. N. & Häusermann D. Two-dimensional detector software: from real detector to idealized image or two-theta scan. *High Pressure Research* **14**, 235-248 (1996).
5. Schneemann, A. *et al*. Different breathing mechanisms in flexible pillared-layered metal–organic frameworks: impact of the metal center. *Chem. Mater.* **30**, 1667-1676 (2018).
